# Solitary fibrous tumor of the kidney: A case report

**DOI:** 10.1016/j.ijscr.2019.08.004

**Published:** 2019-08-20

**Authors:** Selim Zaghbib, Marouene Chakroun, Mohamed Ali Essid, Ahmed Saadi, Abderrazak Bouzouita, Amine Derouiche, Mohamed Riadh Ben Slama, Haroun Ayed, Mohamed Chebil

**Affiliations:** Department of Urology, Charles Nicolle Hospital, Tunis, Tunisia

**Keywords:** Solitary fibrous tumor, Rare renal tumor

## Abstract

**Introduction:**

Solitary fibrous tumor (SFT) is rare, representing 2% of all soft tissue tumors and is usually described in the thoracic cavity. Only 105 cases of renal SFT have been reported.

**Case presentation:**

A 55-year-old men with lower urinary tract symptoms, had a routine renal ultrasound which showed a cortical mass of the left kidney measuring 36 × 23 × 39 mm, with peripheral enhancement, and a central fluid collection on CT. On MRI, it was iso-intense to the kidney in T1 and hyper-intense with restricted diffusion in T2 images. The diagnosis of renal cell cancer was likely and an open partial nephrectomy was performed. Microscopic examination showed a mesenchymal neoplasm with long spindle cell and Immunohistochemical staining positive for CD34 and Bcl-2 confirming the diagnosis of SFT.

There was no evidence of tumor recurrence or metastasis nine months after discharge.

**Discussion:**

Up to now, only 105 cases of occurring renal SFT have been reported. SFTs are usually asymptomatic when they have a small size, that’s why the diagnosis is often delayed. Blood tests do not have any diagnostic value. Imaging features are not specific for the diagnosis of SFT which are diagnosed as renal cell carcinoma and treated as such. Typical immunohistochemical characteristic is a high positivity for CD34. As SFT has a malignant potential, careful follow-up is mandatory,searching for local recurrence or metastasis which was reported in few cases.

**Conlusion:**

SFTs are indolent tumors and are usually diagnosed as renal cell carcinomas preoperatively and the final diagnosis is always based on immunohistochemical study.

## Highlights

• Solitary fibrous tumor is a rare entity, representing 2% of all soft tissue tumors.
• The disease is usually described in the thoracic cavity, only 105 cases of renal solitary fibrous tumor have been reported.
• Solitary fibrous tumors are usually asymptomatic thus they are diagnosed as renal cell carcinoma and treated as such.
• Typical immunohistochemical characteristic is a high positivity for CD34.
• As solitary fibrous tumor has a malignant potential, careful follow-up is mandatory.

## 1 Introduction

Solitary fibrous tumor (SFT) is a rare entity, representing 2% of all soft tissue tumors [1]. Histologically, SFT displays an hemangiopericytomalike growth pattern and immunohistochemical staining for CD-34 and Bcl-2 which are considered as markers of those tumors [2]. The disease is usually described in the thoracic cavity, yet it may concern other sites including the kidney. Up to now, only 105 cases of renal SFT have been reported. We report a new case of solitary fibrous tumor of the kidneys fortuitously found in a 55-year-old patient and treated by nephron-sparing surgery. Our work has been reported in line with the SCARE criteria [3].

## 2 Case presentation

**Fig. 1:**
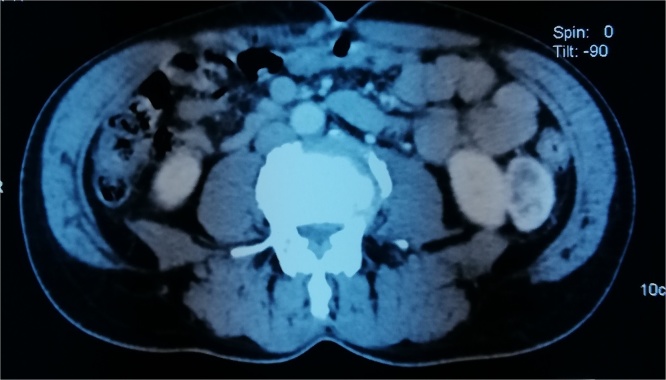
CT revealing an exophytic lesion in the lower pole of the left kidney, measuring 36 × 23 × 39 mm, well demarked, with peripheral enhancement, and a central fluid collection.

**Fig. 2:**
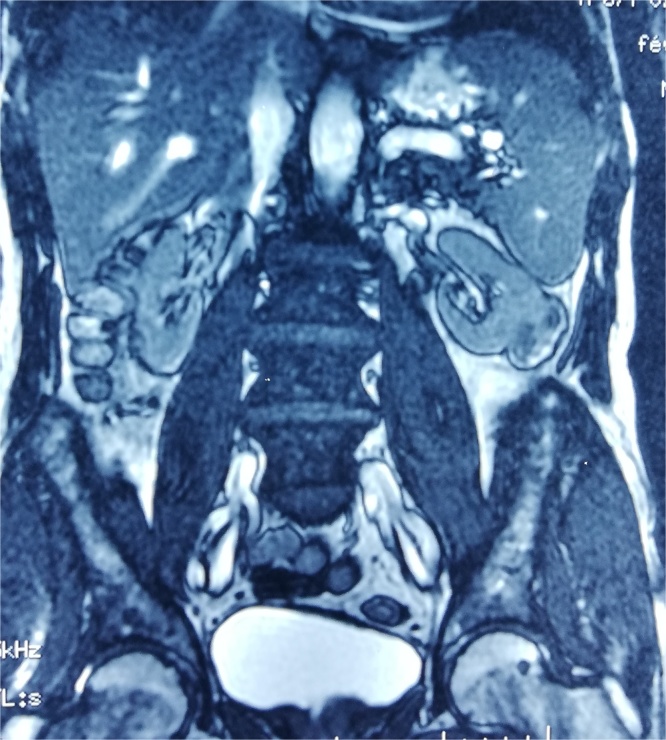
MRI-T2 weighted image demonstrating a hyper intense peripheral signal associated to an exocentric, heterogenous and irregular hypointense signal in the center of the tumor.

A 55-year-old male, with no past medical history, was referred to our department for lower urinary tract symptoms. The patient has neither pain nor hematuria. Physical examination and blood tests were normal. Cytobacteriological examination of the urine was sterile. A routine renal ultrasound (US) showed a 36 mm cortical mass on the low pole of the left kidney with no dilation or deformation of the renal pelvis calyces. The ureters were normal. Subsequent computed tomography (CT) revealed an exophytic lesion in the lower pole of the left kidney, measuring 36 × 23 × 39 mm, well demarked, with peripheral enhancement, and a central fluid collection (Fig. 1). No intratumoral calcification was identified. No invasion of perinephric fat tissues or adjacent structures, such as renal vein or inferior vena cava (IVC) was noted. No metastasis or lymphadenopathy was evident. On magnetic resonance imaging (MRI), the mass was iso intense to the kidney in T1 weighted image and hyper intense with restricted diffusion in T2 weighted image. T2 weighted image also demonstrated a hyper intense peripheral signal associated to an exocentric, heterogenous and irregular hypointense signal in the center of the tumor (Fig. 2).

The diagnosis of renal cell cancer was very likely, the patient underwent an open surgery. It was an 80% exophytic tumor lying on the lower pole of the left kidney, a clampless partial nephrectomy was performed. The postoperative course was uneventful and the patient was discharged on the fourth postoperative day.

Laboratory examination showed a well circumscribed, white, firm tumor confined to the lower pole which measuring 4 × 4 × 3 cm. There was no macroscopic capsular involvement.

**Fig. 3:**
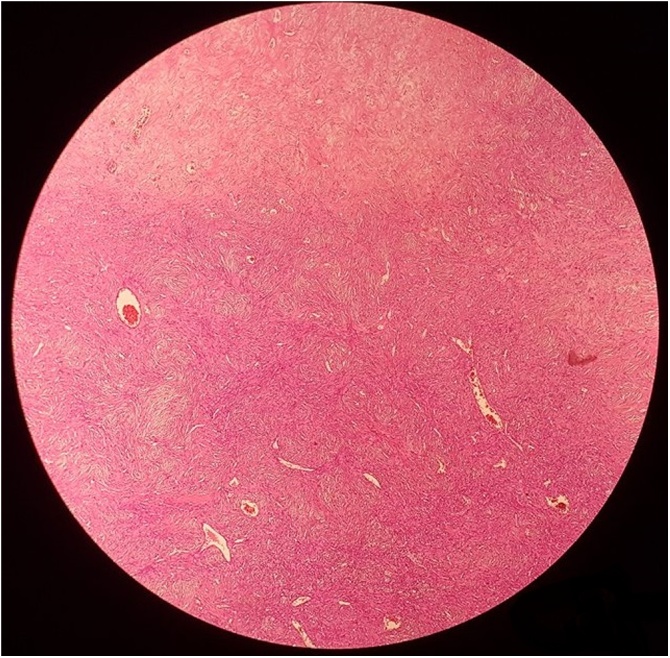
HEx40 : Proliferation composed of long spindle cell with acidophilic cytoplasm and vesicular nuclei, showing a patternless architecture with irregularly branching hemangiopericytoma-like vessels.

**Fig. 4:**
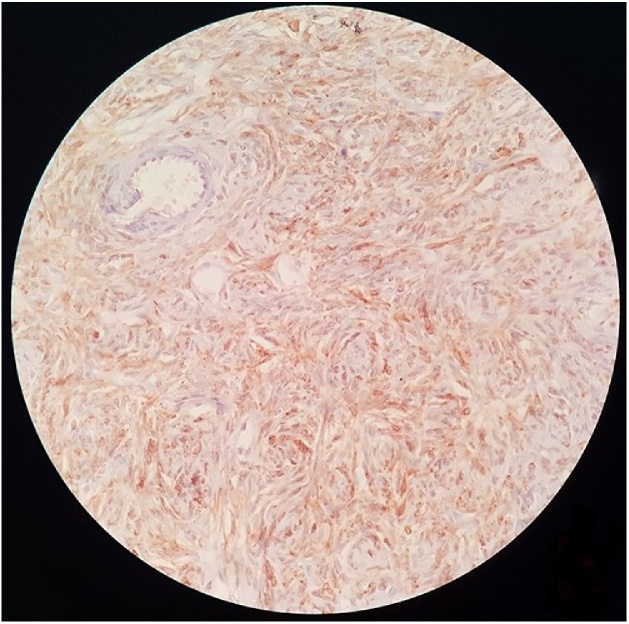
Immunohistochemical staining positive for CD34.

Microscopic examination showed a well mesenchymal neoplasm surrounded by fibrous tissue occasionally separated by strip-like bands of collagen. The proliferation was composed of long spindle cell with acidophilic cytoplasm and vesicular nuclei, round to oval, organized in a patternless architecture with a combination of alternating hypocellular and hypercellular areas separated from each other by thick bands of hyalinized collagen. It also showed a thin-walled, hemangiopericytoma-like vessels (Fig. 3). Mitotic activity and atypia have not been observed. Immunohistochemical staining was positive for CD34 and Bcl-2 and HMB45 stain was negative (Fig. 4). Based on the histological and immunohistochemical features, the diagnosis of SFT of the kidney was established.

Nine months after discharge the patient had a Chest-abdomen-pelvis CT follow up with no evidence of tumor recurrence or metastasis.

## 3 Discussion

In 1931, SFT was firstly reported as a tumor of the pleura. Although SFT is commonly thought of as an intrathoracic tumor, it could arise from extra thoracic organs, including the kidney [4]. Up to now, only 105 cases of occurring renal SFT have been reported. Review of the literature shows that mean age at diagnosis is 52.8 years [5] and that the tumor is usually fortuitously diagnosed. Clinical symptoms could be a palpable mass, a flank pain and often hematuria but SFTs are usually asymptomatic when they have a small size, that’s why the diagnosis is often delayed. Blood tests do not have any diagnostic value. Hypoglycemia can be found in some sites as a paraneoplastic syndrome, but never for kidney SFTs. Although, imaging is useful for the evaluation of extension and recurrence of renal tumors, CT, US and MRI features are not specific for the diagnosis of SFT. That’s why SFT cases were usually diagnosed as renal cell carcinoma and treated as such. Thereby, most reported patients underwent a radical nephrectomy and few cases had nephron-sparing surgery or tumor biopsy [5].

Surgical resection is the standard treatment and complete resection can be associated with a favorable prognosis, even if the SFT is histologically diagnosed as malignant.

Histologically, SFTs are distinguished by a hypercellular stroma of bland spindles cells with no pattern architecture [2]. Typical immunohistochemical characteristics are a high positivity for CD34 – regarded as an indispensable finding in the diagnosis [2] and to a lesser extent for Bcl2, vimentin and CD99. It’s remarkable that criteria of malignancy were proposed by some authors, since SFTs are considered as intermediate malignant tumors, including histological criteria such as increased cellularity with crowded/overlapping nuclei, cellular pleomorphism, mitotic count of more than 4 per 10 high-power fields, presence of necrosis and negativity in CD-34 and Bcl-2 and clinical features as a large tumor size and an extra thoracic location. Actually, Fine et al described a case of malignant renal SFT negative CD-34, which developed distant metastasis after surgery [6].

As SFT has a malignant potential, careful follow-up is mandatory, searching for local recurrence or/and distant metastasis which was reported in few cases [5].

## 4 Conclusion

In conclusion, we reported a new case of a SFT in an unusual location (kidney). It was diagnosed preoperatively as renal cell carcinoma and treated as such, as its diagnosis is based on immunohistochemical study. Although our case showed no evidence of recurrence or distant metastasis, SFT has a malignant potential and careful follow-up is necessary.

## Ethical approval

Charles Nicolle Teaching Hospital ethic committee, Tunis, Tunisia.

## Funding

No source of funding.

## Consent

Written informed consent was obtained from the patient for publication of this case report and accompanying images.

## Author contribution

**Zaghbib S**: concept or design, data collection, data analysis or interpretation, writing the paper.

**Chakroun M**: concept or design, data collection, data analysis or interpretation, writing the paper.

**Essid MA**: data collection, data analysis or interpretation, writing the paper.

**Saadi A**: data collection, data analysis or interpretation.

**Bouzouita A**: data collection.

**Derouiche A**: data collection.

**Ben Slama MR:** data collection.

**Ayed H:** data collection.

**Chebil M**: writing the paper.

## Guarantor

Selim Zaghbib.

## Registration of research studies

This is no research study.

## Provenance and peer review

Not commissioned, externally peer-reviewed.

No financial and personal relationships with other people or organisations that could inappropriately influence (bias) their work.

## Declaration of Competing Interest

No financial and personal relationships with other people or organisations that could inappropriately influence (bias) their work.
